# Ceramic Spheres—A Novel Solution to Deep Sea Buoyancy Modules

**DOI:** 10.3390/ma9070529

**Published:** 2016-06-29

**Authors:** Bo Jiang, Gurdial Blugan, Philip N. Sturzenegger, Urs T. Gonzenbach, Michael Misson, John Thornberry, Runar Stenerud, David Cartlidge, Jakob Kuebler

**Affiliations:** 1 Laboratory for High Performance Ceramics, Swiss Federal Laboratories for Materials Science and Technology, Dübendorf 8600, Switzerland; gurdial.blugan@empa.ch; 2 De Cavis AG, Dübendorf 8600, Switzerland; philip.sturzenegger@decavis.com (P.N.S.); urs.gonzenbach@decavis.com (U.T.G.); 3 Almath Crucibles Ltd., Newmarket, Suffolk CB8 9NE, UK; michael@almath.co.uk; 4 Moulded Foams, Blackwood, Gwent South Wales NP12 2EU, UK; jrt@mouldedfoams.com; 5 Plasto AS, Andalsnes 6300, Norway; Runar@plasto.no; 6 Pera Technology, Melton Mowbray, Leicestershire LE13 0PB, UK; David.Cartlidge@uk-matri.org

**Keywords:** ceramics, thin-walled hollow sphere, deep-water buoyancy modules

## Abstract

Ceramic-based hollow spheres are considered a great driving force for many applications such as offshore buoyancy modules due to their large diameter to wall thickness ratio and uniform wall thickness geometric features. We have developed such thin-walled hollow spheres made of alumina using slip casting and sintering processes. A diameter as large as 50 mm with a wall thickness of 0.5–1.0 mm has been successfully achieved in these spheres. Their material and structural properties were examined by a series of characterization tools. Particularly, the feasibility of these spheres was investigated with respect to its application for deep sea (>3000 m) buoyancy modules. These spheres, sintered at 1600 °C and with 1.0 mm of wall thickness, have achieved buoyancy of more than 54%. As the sphere’s wall thickness was reduced (e.g., 0.5 mm), their buoyancy reached 72%. The mechanical performance of such spheres has shown a hydrostatic failure pressure above 150 MPa, corresponding to a rating depth below sea level of 5000 m considering a safety factor of 3. The developed alumina-based ceramic spheres are feasible for low cost and scaled-up production and show great potential at depths greater than those achievable by the current deep-sea buoyancy module technologies.

## 1. Introduction

As available oil and gas reserves that are easily and affordably extracted are becoming depleted, exploitations are continuously moving towards deeper water (>3000 m) in harsh environments. The discovery of new oil/gas reserves in deep or arctic waters has accounted for 41% of new reserves discovered, and represents a very exploitable market for the offshore oil and gas industry [1]. Buoyancy modules are required for the components and systems of offshore extractions such as drill risers and gas exploration lines to maintain their desired position under the sea. These units are currently made from syntactic foams reinforced with micro-spheres and macro-spheres [2,3,4,5,6]. For example, the buoyancy modules containing glass hollow microspheres with a density of 0.3 g/cm^3^ can achieve a crush strength of 41 MPa, corresponding to a rating depth of 2180 m below sea water [7]. The modules containing polymer macrospheres usually can achieve compressive strength of 30–40 MPa such as the MacroFoam 30 from Engineered Syntactic Systems (ESS), corresponding to a depth rating of 2000 m [8]. When working in deep sea at depths of up to 5000 m, the water pressure reaches above 50 MPa. The currently used buoyancy module technologies are unsuitable owning to their limited mechanical strength as well as manufacturing inconsistences. When they fail, the repair is extremely expensive, at approximately €100,000 with considerable additional loss of production costs per day, not to mention the potential environmental damage.

Therefore, we propose buoyancy units that are fabricated using ceramic macrospheres (40 ± 10 mm) instead of plastic and glass ones, to develop a final product with greatly enhanced hydraulic failure pressure as well as acceptable buoyancy properties. Such products have been targeted for a continuous operation below sea level at depths of >3000 m. The fabrication of ceramic hollow macrospheres for deep-sea applications can be dated back to military applications in the 1960s [9]. For example, porcelain or alumina based ceramic spheres have been developed using a roto-casting or gel-casting process for deep-sea applications; however, their slow fabrication process and high production cost (e.g., $550 USD per sphere ) are not feasible with the mass production requirement of buoyancy modules in the range of several millions of pieces per year [10,11]. It is a challenge to produce “perfectly” shaped hollow thin-walled ceramic macro-spheres with the required buoyancy and mechanical properties in an economic way.

In this work, we have developed prototypes of ceramic thin-walled hollow macrospheres, CeraSphere™, after comprehensive studies on material selection and the geometric designs. Various characterizations were carried out to investigate such ceramic spheres in terms of structure, sphericity, buoyancy and mechanical properties. The isostatic compressive strength of the ceramic hollow spheres has been considered the most critical product criterion, as they could burst due to over pressure and result in implosions during service [6,12]. Hence, a proof pressure test has been deployed in this study and has demonstrated that the ceramic spheres successfully withstand a short-term pressurization without any failure or implosion. Together with the sphere density information, we have compared the compressive burst strength of our developed ceramic spheres with other commercial syntactic foam buoyancy materials, showing that the ceramic spheres have a great potential as a replacement of the current buoyancy materials due to their excellent buoyancy and mechanical properties.

## 2. Design

The performance specifications of buoyancy modules require (a) high sphere compressive burst strength; (b) low density; (c) high hydrostatic creep resistance; (d) zero water absorption; (e) temperature/chemical stability when setting in syntactic foam resistance during syntactic foam manufacturing (~150 °C) and (f) feasibility for high volume productions. Although ceramic materials are brittle, they exhibit particularly high compressive strength and stiffness in comparison with metals and polymers [13,14]. The creep deformation in ceramics generally becomes noticeable at high temperature, usually >40%–50% of the melting point. In our application, the concerns of hydrostatic creep in the ceramic sphere are rather negligible, as the working temperature is below the ambient temperature [15]. On the other hand, an advantage of using ceramic spheres is their temperature resistance, usually up to 1000 °C; it allows easy handling when adding a syntactic epoxy foam mixture to the hollow spheres in the production of the buoyancy modules, which results in an exothermic reaction up to 200 °C. Moreover, the density of our sintered ceramics is above 98% of the theoretical material density; therefore, no pores exist, which is largely determined by the quality of the raw materials and the forming techniques, showing very good hermeticity against sea water. Overall, both compressive burst strength and buoyancy properties are considered to be the most critical criteria for the ceramic sphere in the determination of the ceramic material choice as well as geometric design.

**Figure 1:**
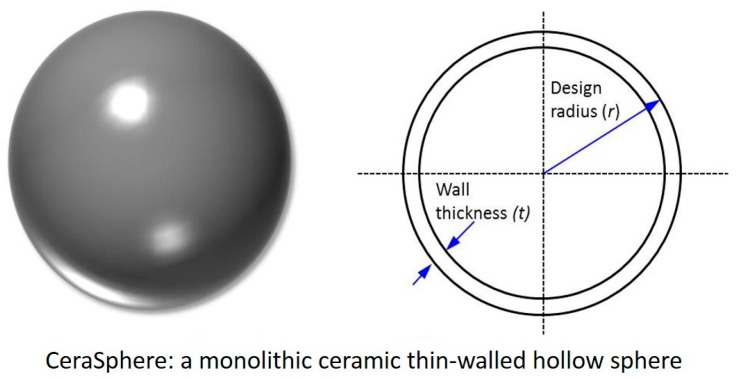
A concept schematic of the ceramic sphere.

The concept schematic of the ceramic sphere is illustrated in Figure 1 with geometric parameters: design radius (*r*) and wall thickness (*t*). A 25 mm radius was chosen for the prototyping in this study. The hydrostatic compressive burst strength criterion for the ceramic sphere is 83 MPa, as specified by the industrial end users, corresponding to a deep sea depth rating of 3660 m based on a safety factor of three. This safety factor has been used in the entire investigation for the prediction of the safe operational depth. The burst strength as well as wall thickness of ceramic spherical shells can be estimated by using elastic buckling theory [16]. Thus, the minimal wall thickness of the ceramic sphere can be calculated using Equation (1) for estimating material failure, in which *σ* is the compressive strength, *t* is the wall thickness of the ceramic sphere, *r* is the spherical radius, and *P_m_* is the hydrostatic pressure. With the estimated maximum wall thickness, sphere density (*ρ_sphere_*) of the ceramic spheres can be confirmed using Equation (2), where *ρ* is the material density. Considering a spherical shell shape, the elastic buckling pressure of the ceramic sphere under pressure can be estimated by Equation (3), where *E* is the Young’s modulus, *ν* is the Poisson ratio and *P_CR_* is the elastic buckling pressure. *P_CR_* must be larger than *P_m_* for the ceramic sphere’s mechanical stability and integrity. These engineering calculations are simple and efficient tools for our initial assessment for the material selection and sphere geometric designs at the stage of prototyping. These calculations are rather limited. A more accurate and detailed comprehensive numerical modeling on the mechanical studies of the ceramic spheres is being performed and will be reported in a future work:

(1) $$P_{m}=\frac{2\sigma \times t}{r}$$

(2) $$\rho_{sphere}=\frac{m}{v}=\frac{\rho \left(r^{3}-{\left(r-t\right)}^{3}\right)}{r^{3}}$$

(3) PCR=2×E(rt)23×(1−ν2)

**Figure 2:**
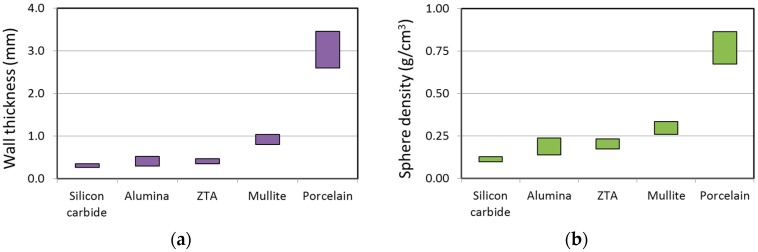
Comparisons of calculated (**a**) wall thickness and (**b**) sphere density of the hollow sphere with a radius of 25 mm made from various engineering ceramic materials that can theoretically withstand 105 MPa of compressive burst pressure. ZTA = zirconia toughened alumina.

Using the above equations, both wall thickness and sphere density of ceramic spheres made from common engineering ceramic materials in Table 1 can be estimated and compared under the required hydrostatic pressure (see Figure 2).

Undoubtedly, non-oxide ceramics such as silicon carbide (SiC) have low density and excellent mechanical properties. The required wall thickness of a ceramic sphere made of SiC is as thin as only 0.27–0.35 mm and the correlated sphere density corresponds to 0.10–0.13 g/cm^3^, showing very good material properties for producing ceramic spheres. However, their difficult and expensive processing—sintering at extremely high temperatures (>1800 °C) and in a controlled inert gas atmosphere presents unsurpassable economic challenges for high volume production. The use of non-oxide ceramics becomes very questionable for this work. Silicate materials normally have a low ceramic processing temperature, typically below 1350 °C, which is favorable for economic mass production. However, because of their low compressive strength, silicate materials such as porcelain require a minimal wall thickness of 2.60 mm in order to meet the compressive strength criterion. The resulting sphere density is rather high (0.67–0.86 g/cm^3^), making them unsuitable from a buoyancy prospective (density <0.5 g/cm^3^). Oxide ceramics such as ZTA and mullite offer good mechanical properties for a ceramic sphere: the estimated sphere density of the ceramic sphere made of ZTA is about 0.23–0.17 g/cm^3^ for withstanding the required hydrostatic pressure. But sintering temperatures of ZTA and mullite are usually greater than 1600 °C and their raw material cost is relatively high, posing high production costs, which is not favorable for the mass production. Overall, we have identified that alumina ceramics are the most optimal material for producing ceramic spheres, because of their (i) good minimal wall thickness (0.30–0.52 mm); (ii) desired sphere density (0.14–0.24 g/cm^3^) under the required pressure; and (iii) feasible sintering process that can be lowered further by introduction of additive oxides [21,22].

**Figure 3:**
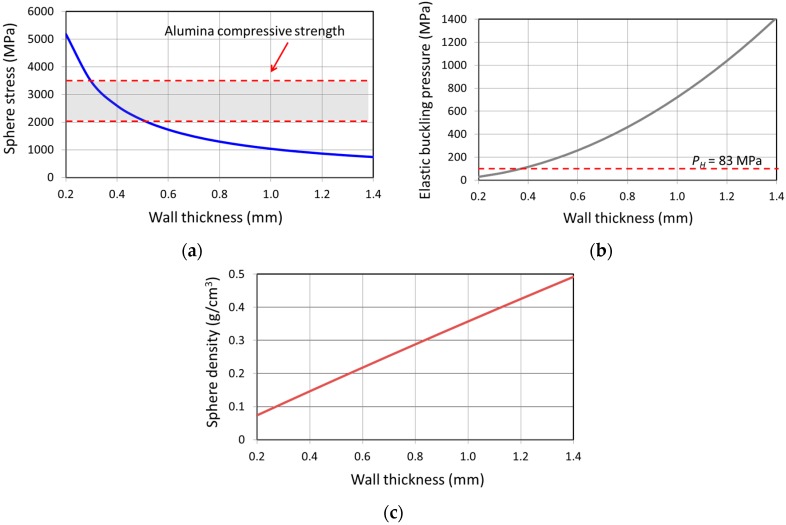
(**a**) calculated sphere stress at 83 MPa of hydraulic pressure; (**b**) elastic buckling pressure of the sphere walls and (**c**) sphere density for the alumina-based ceramic spheres made of alumina materials with various wall thicknesses.

Additionally, we have calculated material failure stress, elastic buckling pressure and sphere density (see Figure 3) of an alumina-based ceramic sphere (25 mm in radius) with various wall thickness at the hydrostatic compressive pressure criterion (83 MPa). The material failure stress was calculated using Equation (1). These calculations provided us a good basis for the design of wall thickness with considerations of both mechanical and buoyancy properties (see Figure 3). It has been calculated that the material failure stress is lower than the alumina material compressive strength for a ceramic sphere with wall thickness greater than 0.5 mm. For a ceramic hollow sphere, an elastic buckling pressure higher than the designated hydrostatic compressive pressure (83 MPa) is required, which actually has been calculated to be >115 MPa for the sphere with a wall thickness >0.4 mm. On the other hand, the allowable wall thickness for the ceramic sphere with less than 0.5 g/cm^3^ of sphere density lies in the range of 0.5–1.4 mm (see Figure 3). Therefore, we conclude that the minimal wall thickness is about 0.5 mm for an alumina ceramic sphere with a radius of 25 mm with acceptable buoyancy and mechanical criteria.

## 3. Results

**Figure 4:**
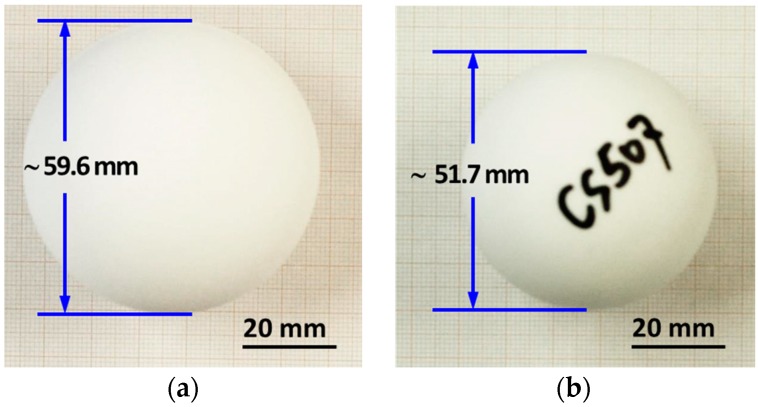

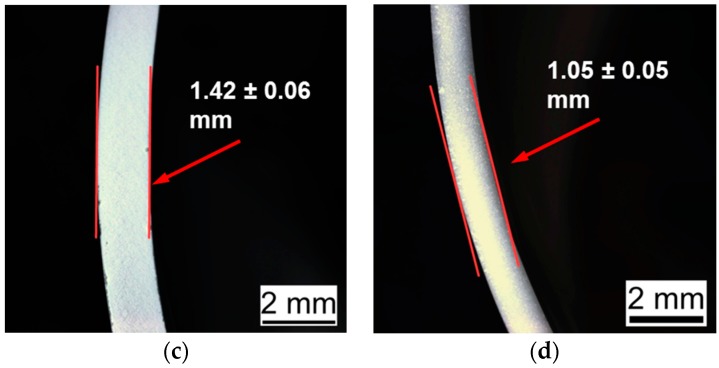
Images of (**a**,**c**) for a green-state CS T10 sample with its cross-section image and (**b**); and (**d**) a sintered CS T10 sample with its cross-section image.

Ceramic spheres were produced by using varied amounts of slip (25 g, 30 g and 40 g) that were designated as CS T05, CS T07 and CS T10, respectively, which achieved the same dimension and densification but different wall thicknesses. Figure 4 reveals the dimensional comparison of ceramic spheres (CS T10) in green and sintered states, showing a diameter of 59.6 mm in the green and 51.7 mm as sintered. The sphere dimensional shrinkage was estimated to be about 13% (see Figure 4a,b). There was no cracking observed in the green ceramic sphere, revealing that a proper molding and drying process was achieved without any structural failure due to the sphere shrinkage. The cross-section of the samples shows their wall thicknesses in the green and sintered states: the green ceramic sphere has an average thickness of 1.42 ± 0.06 mm (see Figure 4c); the sintered one has a wall thickness of 1.05 ± 0.05 mm, indicating a firing shrinkage of 26% (see Figure 4d). At the sphere’s cross-section, we observed that the sintered ceramic sphere achieved very thin and dense spherical walls (see Figure 4d). The observed material density of the sintered ceramic sphere was about 3.88 ± 0.02 g/cm^3^, corresponding to 98% of alumina’s theoretical density.

**Figure 5:**
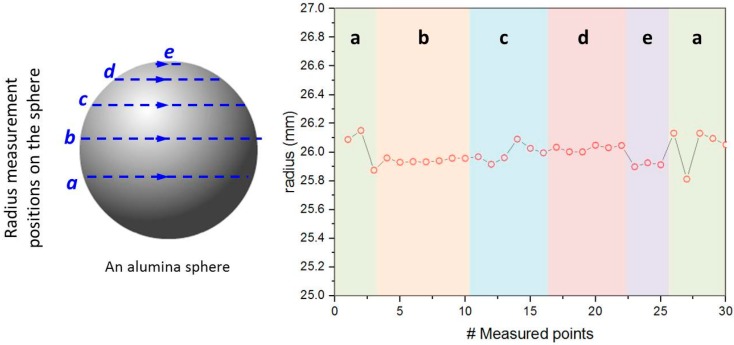
Radius measurement results of an alumina sphere, where the blue dotted lines are the measured areas in the sample.

The radii results measured on a CS T10 ceramic sphere’s surface are presented in Figure 5, in which the measured points are indicated as circular dotted lines in blue. The ceramic sphere has an average radius of 26.00 mm with a standard deviation of 0.10 mm:

(4) $$B=\frac{\rho_{water}-\rho_{Cerasphere}}{\rho_{water}}$$

**Figure 6:**
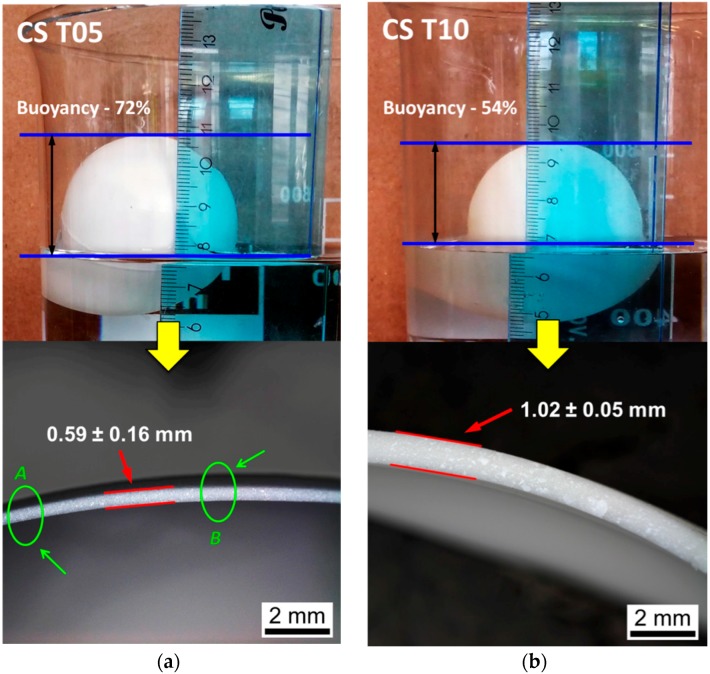
Buoyancy tests in simulated sea water and wall thickness results for the alumina based ceramic sphere: (**a**) CS T05 and (**b**) CS T10.

Figure 6 shows two types of fabricated samples, labeled as CS T05 and CS T10, which are tested in simulated sea water for their buoyancy properties. All spheres were floating in the water with more than 50% of their volume above the water. The results demonstrate that these alumina based ceramic spheres have very promising buoyancy performance. For example, as calculated by Equation (4), the CS T05 with a wall thickness of 0.59 ± 0.16 mm achieved a buoyancy factor *B* of 72% (see Figure 6a), while the corresponding sphere density was about 0.47 g/cm^3^ as calculated by Equation (2). The wall thickness indicated in the figure was measured at the cross-section of the fractured sample. The obtained deviation reveals the high level of uniformity of the wall thickness in the samples, which is critical to the mechanical performance of the ceramic sphere [23].

**Figure 7:**
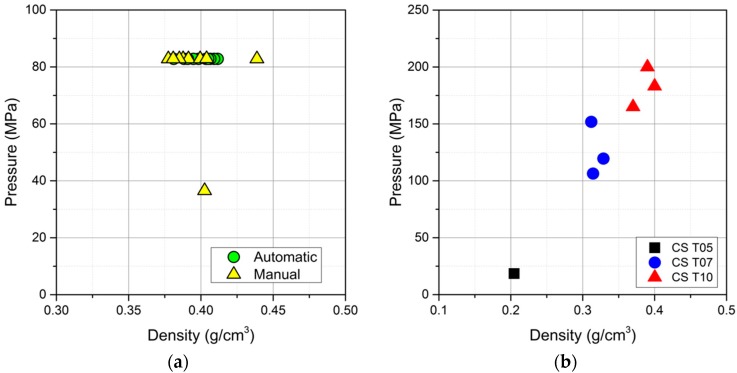
(**a**) proof test results at isostatic compressive pressure (83 MPa) for alumina CS T10 ceramic spheres (* sphere imploded); (**b**) a failure pressure test for the alumina ceramic spheres with various wall thicknesses (CS T10, CS T07 and CS T05).

Figure 7a reveals the isostatic pressure proof test results of 20 samples produced via an automatic rotation process and nine samples produced using a manual casting manner. In this test, the sample was placed into the hydrostatic pressure chamber. The chamber was then sealed and an isostatic pressure applied up to 83 MPa with a ramp rate of 16.6 MPa/min, dwell for 10 min, and then released to the ambient conditions. All 20 spheres made by the automatic rotation process are able to sustain 83 MPa without any implosion; on the other hand, one out of nine samples produced by manual rotation failed, giving a yield of 89%.

Moreover, samples with various wall thicknesses have been evaluated with regard to their hydraulic failure pressure. Although the number of tested samples is limited for a statistical observation, the authors still believe that these results can serve as preliminary information for comparison purposes with other hollow sphere products. As shown in Figure 7b, the CS T10 achieved 182.7 ± 14.3 MPa of average failure pressure, while the CS T07 achieved 125.9 ± 19.1 MPa.

## 4. Discussion

Comparing the dimension of the green and sintered spheres (see Figure 4), it was observed that the sphere dimensional shrinkage is much smaller than the sphere wall shrinkage. This shrinkage difference is very likely caused by the thin-wall hollow spherical shape; the densification of alumina that shrank from both outer and inner surfaces to each other during sintering might hinder the sphere shrinking from its outer radius. Nevertheless, more details of this shrinkage difference in hollow shaped ceramic objects will be investigated in our future work.

In our investigation, the sphericity was evaluated in terms of radius characterization on a single ceramic sphere (see Figure 5). Uniform radii results indicate a good sphericity. The system error in our radius measurement is about ±0.01 mm. Such a radius deviation (<0.4%) is considered to be small and the sample is very close to a perfect sphere. Similar results have been found in different sphere batches. It is generally believed that the sphericity of a hollow ceramic sphere is critical to its mechanical performance, e.g., hydraulic failure pressure. Poor sphericity can introduce irregular local radius of curvature and therefore develop stress concentrations on the sample under isostatic compressive pressing, eventually leading to mechanical failure [23]. It is believed that the quality of the casting mold, forming process as well as sintering are governing the sphericity quality of the ceramic sphere. The physical dimension of the molds must be very precise in order to cast a perfect spherical shape. A proper forming process assures that the ceramic content can be deposited on the inner surface of the molds uniformly and form a good green spherical shape inside the molds so as to avoid any formation of irregular wall thickness as well as spherical curvature. Last but not least, a sintering process that produces dense ceramic materials, which must be carried out in a proper way, so that the shrinking of ceramic materials occurs evenly over the entire object without any dimensional deformation.

Furthermore, the wall thickness of the ceramic sphere was modified by using lower amounts of ceramic slip for the formation of the green spheres. As the wall thickness of the sample decreased from 1.02 mm to 0.59 mm, it was shown that the corresponding buoyancy factor increases from 54% to 72% (see Figure 6 and Table 2). Certainly, thinner wall thicknesses, can deliver greater buoyancy performance in the ceramic sphere. However, one must be aware of the limitation in the achievement of thin walls because too low a quantity of ceramic slip can cause uneven deposition of ceramic slip in the molds and hence inhomogenous wall thickness, which was observed in CS T05 samples. For example, as shown in Figure 6a, the wall thickness in CS T05 at point A and B can differs by 0.12 mm. It was observed that the maximum deviation of wall thickness in CS T05 was as high as 0.16 mm due to non-uniform formation of spheres.

The mechanical performance of the ceramic sphere has been evaluated in two ways with respect to its deep-sea application: an isostatic compressive pressure proof test and a failure pressure test. Both are industrial standard test methods and are generally considered to be the most efficient methods for validating the design of prototypes. Although some other non-destructive proof tests are used in industry for qualifying buoyancy modules at a given pressure rating [12], we mainly used the ceramic sphere CS T10 samples for the pressure proof test. As previously mentioned, the applied isostatic pressure (83 MPa) corresponds to a depth rating of 3660 m below sea level with a safety factor of 3. This depth rating is considered as the desired operating depth defined by end users in the oil exploitation industry. The authors believe that such a test can serve as an efficient evaluation tool for a quality check of the produced ceramic spheres as well as the predication of any implosions that may be caused either by material failure or elastic instability, which can be easily adapted in the quality control in future mass production.

It was determined that the single sphere which failed and was produced by the manual casting approach, was due to structural defects in the sample that were formed during the manual rotation casting process. Clearly, these ceramic spheres, sintered at 1600 °C with wall thickness of ~1.0 mm have proven to be mechanically stable at the required isostatic compressive pressure. In particular, we have found that 100% of the alumina ceramic spheres produced by the automatic casting process have passed the proof test, showing the advantage of automated over manual production in terms of reliability. It is clear that the formation process of the green ceramic sphere is one of the most critical fabrication steps and largely governs the mechanical performance of the end-products.

Based on the results of the failure pressure test, with a safety factor of 3, the corresponding depth rating for the CS T10 and CS T07 ceramic spheres is 5450 m and 4630 m, respectively. On the other hand, the CS T05 shows a much lower hydraulic failure pressure, which is mainly caused by uneven wall thickness due to the low slip volume to mold surface ratio. Nevertheless, based on our knowledge, it is the first time that alumina hollow spheres with a sphere density of 0.3–0.4 g/cm^3^ are able to achieve such high isostatic compressive pressure as well as outstanding depth rating.

**Figure 8:**
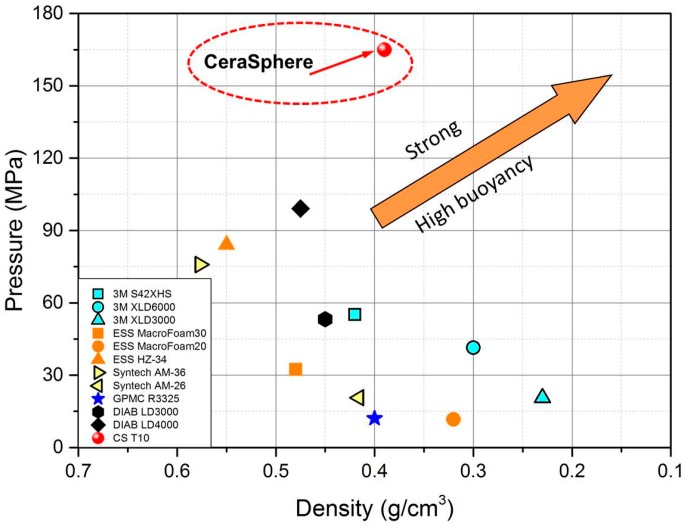
A benchmark comparison of the ceramic sphere and other commercial buoyancy products in terms of hydrostatic compressive burst strength and density [24,25,26,27,28,29].

The state-of-the-art buoyancy modules are traditionally made of syntactic foams reinforced with hollow polymer macrospheres or glass microspheres. The hollow macrospheres are usually made of epoxy or expanded polystyrene that have similar dimensions (10–100 mm) to our ceramic spheres. We compared our developed ceramic spheres (CS T10) with the main buoyancy products on the market in terms of hydraulic failure pressure and buoyancy density, where the failure pressure of 165 MPa (lowest one for CS T10) was used as the maximum achieved value for the ceramic sphere (see Figure 7b). In an attempt to judge the performance of the developed macroscopic ceramic hollow sphere, pressure ratings of existing buoyancy materials are plotted against their density in Figure 8. For the Cersaphere, the actual sphere density is used, whereas for existing buoyancy materials the density relates to the entire syntactic foam reinforced buoyancy modules, including hollow spheres and epoxy matrix. Because the weight of the syntactic foam is rather negligible, the density of counterpart products can be considered as the density of the glass or polymer hollow spheres themselves. Figure 8 clearly shows that the hydraulic failure pressure of the ceramic sphere is generally 2–3 times higher than that of state-of-the-art products. Although some alternative technologies have lower density and better buoyancy than the ceramic sphere, their hydraulic failure pressure is comparatively low and can only survive a depth rating of 2000 m below sea. For an ideal hollow sphere for use in deep-sea buoyancy modules, it is required to have high mechanical strength as well as low sphere density. Our developed ceramic sphere is able to achieve a density of 0.39 g/cm^3^ in combination with an isostatic compressive pressure above 150 MPa.

The advantages of using ceramic hollow spheres are not limited to their mechanical and buoyancy properties. Thanks to the material stability of ceramics, the developed ceramic spheres have better temperature resistance and thus can speed up the manufacturing of syntactic foams, which is an exothermic process and requires a staged filling process of hollow polymer macrospheres. Overall, the developed alumina based ceramic spheres have demonstrated, excellent potential as a promising replacement of hollow polymer-based macrospheres and glass microspheres for developing buoyancy modules in deep-sea offshore drilling applications.

## 5. Materials and Methods

Ceramic spheres have been produced in molds, which were made up of two well-fitted hemispheres. First, a water-based slurry containing 40 vol. % of >99.7% grade alumina powder was poured into the molds. Various amounts of slip (25 g, 30 g and 40 g) was loaded into the molds for achieving ceramic spheres with different wall thicknesses. The samples were designated as CS T05, CS T07 and CST10, respectively. Second, by rotating the molds, the alumina suspension was uniformly deposited onto the inner surface of the molds to develop a green thin-layered hollow sphere. Next, the produced green spheres were removed from the molds, dried at room temperature for several hours, and sintered at 1600 °C to full densification.

Various material and performance characterizations have been carried out for the alumina based ceramic spheres. To evaluate the wall thickness, the sample was firstly broken and the cross-section of the fracture is observed under optical microscopy (ZEISS Discovery V20, Carl Zeiss AG, Oberkochen, Germany) for the wall thickness measurement. The material density was measured by means of Archimedes’ principle. The sphericity of the produced ceramic spheres was evaluated in terms of radius variation with the use of a coordinate-measurement machine (DEA Gamma 0101, Hexagon Metrology AG, Unterentfelden, Switzerland). By recording the *x*-, *y*- and *z*-coordinates of points on the surface of a sphere precisely, the center of the sphere and its radius at each point were measured and calculated. The standard deviation of the measured radii is taken as a criterion for sphericity quality. The buoyancy performance of the produced ceramic spheres were characterized in three ways: first, the sphere density, *ρ_sphere_*, was calculated by measuring volume as well as weight of the sample by using Equation (2); second, a visual inspection was carried out on the sample which was placed in simulated sea water, a mixture of deionized water and 3.5 wt % sodium chloride; third, the buoyancy performance of the sample was characterized by the buoyancy factor *B* as defined by Equation (4) [30].

## 6. Conclusions

In this work, we have successfully developed ceramic based hollow thin-walled spheres (ceramic spheres) for making buoyancy modules for deep sea (>3000 m) oil exploitation. The product strength calculation has estimated that alumina ceramic can be an optimal material candidate for economically producing ceramic spheres with a radius of 25 mm. An automatic rotational casting process has been developed for producing ceramic sphere green bodies using hemisphere molds with good structural uniformity. These ceramic spheres were successfully produced after a sintering process at 1600 °C. Characterization tests have been carried out to show that the developed alumina based ceramic spheres have achieved a buoyancy of 54% and a sphere density of 0.39 g/cm^3^, for the spheres with a wall thickness of 1.02 ± 0.05 mm. The radius deviation in a single ceramic sphere is rather small, indicating a good sphericity. In terms of mechanical performance, 100% of 20 ceramic spheres made by an automatic rotation casting process pass the pressure proof test at 83 MPa, which corresponds to a depth rating of 3660 m with a safety factor of 3. More importantly, these ceramic spheres have achieved a maximum hydraulic failure pressure as high as 182.7 ± 14.3 MPa, showing that they can be operated at greater than 5000 m below sea level. Moreover, our ceramic spheres are made from low cost materials with a robust forming process, and, therefore, upscaling the production process to the relevant volumes appears to be realistic. The spheres cost about 1% of the previous reported product price. Based on our benchmark results, it clearly shows the great potential of the alumina ceramic spheres as an efficient and economic replacement of the currently used hollow polymer or glass spheres for producing buoyancy modules for deep sea exploitation.

## Figures and Tables

**Table 1:** Material properties of common engineering ceramic materials [13,14,17,18,19,20].

Properties	Silicon Carbide	Alumina	Zirconia Toughened Alumina (ZTA)	Mullite	Porcelain
Density (g/cm^3^)	3.1	3.9	4.2	2.8	2.4
Compressive strength (MPa)	3500–3900	2000–3500	2200–3000	1000–1300	300–550
Bend strength (MPa)	400–600	300–500	400–1000	150–180	300–550
Fracture toughness (MPa·m^1/2^)	4	4	5–8	2–3	2
Poisson ratio	0.21	0.23	0.23	0.24	0.17
Young’s modulus (GPa)	410	380	360	150	100
Sintering temperature (°C)	>1800	1400–1600	1600–1700	1600	1200–1400

**Table 2:** Wall thickness, sphere density and buoyancy results of the alumina based ceramic sphere with a radius of 25 mm.

Type	Wall Thickness (mm)	Sphere Density (g/cm^3^)	Buoyancy Factor *B* (%)
CS T05	0.59 ± 0.16	0.21	72
CS T07	0.73 ± 0.05	0.32	63
CS T10	1.02 ± 0.05	0.39	54

## Abbreviations

The following abbreviations are used in this manuscript:

SiC	silicon carbide
ZTA	zirconia toughened alumina
